# Understanding Dysphagia Care in Pakistan: A Survey of Current Speech Language Therapy Practice

**DOI:** 10.1007/s00455-023-10633-7

**Published:** 2023-11-25

**Authors:** Rohma N. Akhtar, Nicholas Behn, Sally Morgan

**Affiliations:** 1 Division of Language & Communication Science, Northampton Square, London, EC1V 0HB UK; 2 City University of London, London, UK

**Keywords:** Low- and middle-income countries, Speech language therapy, Swallowing disorders, Feeding, Dysphagia, Eating and drinking

## Abstract

Dysphagia affects individuals across all ages and has pervasive and potentially life-threatening consequences. Individuals with dysphagia are assessed and treated by speech and language therapists (SLTs), however, little attention has been paid to their practices in Pakistan. This study aimed to explore SLTs practices for dysphagia assessment, signs and symptoms observed during evaluation, and management strategies, alongside barriers and facilitators to service delivery in Pakistan. A 45-item survey was distributed to SLTs online. Responses were received from 101 participants and analyzed descriptively, and open-text responses were analyzed using content analysis. Results revealed that 65.3% SLTs worked across the lifespan, and most (79.4%) had dysphagia-related experience of five years or less. SLTs were an active workforce engaged with varying ages, disorders, and settings, yet dysphagia contributed to a small caseload percentage for most. Analyses found informal clinical exams were more frequently used than instrumental assessments. A variety of service provision facilitators were described, such as supportive teams and accessible resources, and responses about barriers revealed gaps in awareness, education, and guidance. This exploratory study presents novel and unexplored data which provides a deeper understanding of dysphagia-related care in Pakistan.

**Supplementary Information:**

The online version contains supplementary material available at 10.1007/s00455-023-10633-7.

## Introduction

### Dysphagia and Associated Consequences

Dysphagia is described as disordered or delayed movement of food/liquid during the oral, pharyngeal, and oesophageal phases of swallowing [1, 2]. In adults and children, dysphagia may be attributed to congenital or acquired, neurological, cardiorespiratory, metabolic, or inflammatory disorders [3–14]. The true global prevalence of dysphagia is difficult to determine as it is reported in heterogenous disorders and diverse settings. In adults, the prevalence of dysphagia is reported as 11%-55% [15–17], and may be indicative of numerous neurological and non-neurological conditions [9–17]. In children, pediatric feeding disorder, which can include dysphagia, is associated with nutritional, medical, feeding, and/or psychosocial dysfunction [18, 19]. This wider definition can explain the higher reported dysphagia prevalence of 25%-40% in children with typical, and 85–99% with delayed development [3, 19–21]. Dysphagia is associated with a plethora of negative outcomes, including decreased activity and social participation, decline in overall health and quality of life, malnourishment and dehydration, weight loss, aspiration pneumonia, increased hospital stay and mortality [9, 11, 22–29]. Additional consequences of dysphagia include fear of choking, adverse effects on wellbeing, and caregiver stress [30, 31]. Evidence purports that while healthcare professionals may place a greater importance on medical factors, psychological factors are of greater significance to patients [30]. This highlights the importance of rehabilitation management programs to account for patient and family preferences, and the functional impact of dysphagia and its associated problems on daily activities. Therefore, individuals with dysphagia (IWD) may benefit from multidisciplinary teamwork, with emphasis placed on assessment and management by speech and language therapists (SLTs) [32, 33].

### Global Dysphagia Practice

In countries with national SLT registration schemes, e.g., USA, UK, requirements include a recognized bachelor’s or master’s degree in the UK, and a master’s degree in the USA, with demonstrated knowledge of swallowing and communication difficulties, competence in evaluation and intervention, and evidence-based clinical practice, alongside supervised clinical observation for at least nine months [34, 35]. The American Speech-Language-Hearing Association has nearly 200,000 SLT members, approximately 96% female and 55% under 45 years old. Of these, over half work in school settings, 40% in healthcare; additionally, 23% of SLTs work in private practice [34]. In the UK, over 17,000 SLTs are registered as practicing members with the Health and Care Professions Council (HCPC). In a recent survey 53% of SLTs (*n* = 3779) in the UK worked full-time, 96% were female. Of the respondents, 28% worked in community service settings, 27% in the national healthcare system (NHS) and 25% in school settings [35, 36].

Currently in Pakistan, there is no recognized national licensing body for the profession. The workforce comprises SLTs trained within Pakistan and overseas. Pakistani university programs require SLTs to complete a four-year bachelor’s degree or a two-year master’s degree in speech and language therapy (SALT), followed by a year under supervision as a newly qualified SLT. SLTs are involved in dysphagia care across the lifespan, from neonates to elderly individuals, working in independent practice, hospitals, and schools.

### Dysphagia Management

Regarding evaluation, gold standard instrumental assessments are routinely used in high income countries (HICs) to evaluate dysphagia, such as video-fluoroscopic swallow study (VFSS) and fibreoptic endoscopic evaluation of swallowing (FEES) [37]. Other methods may include pulse oximetry, cervical auscultation, and citric acid cough reflex testing, which is an increasingly common practice in the UK particularly with adults [38]; however, there is low-quality evidence that supports their use in isolation [39–41]. While non-standardized means such as clinical swallow exams (CSE) based on observations are in common use globally by SLTs for assessing dysphagia symptoms, the procedural specifications remain unclear, and variability is noted [42–44]. Prior evidence recommends evaluating specific markers during CSE in children, e.g., multiple swallows and wet voice [45–49], and adults, e.g., coughing and difficulty in breathing [50, 51].

Interventions for dysphagia are implemented by SLTs and may include restorative strategies with a direct focus on muscular skill and underlying neurophysiology (e.g., Mendelsohn’s maneuvre and effortful swallow) [52–54], or compensatory strategies (e.g., modifying food/liquid consistencies, and chin tuck posture). Modification of fluid/food consistencies is a common compensatory intervention strategy, though the evidence supporting this practice for children and adults is inconsistent, and disorder-specific, with unclear directions for SLTs [52, 55–60]. Management strategies for adults may include free water protocols, which require careful monitoring and administration [61, 62]. Management plans may also include referrals to psychosocial support groups, with the aims of maintaining the wellbeing of entire family units and IWD, and reducing their stress/burden [33, 63–65].

### Dysphagia in Pakistan

There are a limited number of published studies on dysphagia in Pakistan, with some historic but still potentially relevant due to a slower pace of change in the low-and middle-income country (LMIC) context. This is concerning considering within LMICs, like Pakistan, individuals with disabilities (including feeding difficulties) are at substantially higher risk for undernourishment [66–69]; and pneumonia is a contributing factor to respiratory distress and mortality in adults and neonates [70–73]. Dysphagia may contribute to many of these issues. In adults alone in Pakistan, the prevalence of post-stroke dysphagia is 38%-53% [70, 72], which rises to 84% in adults with post-stroke presenting with chest infections [72].

While instrumental evaluations are standard practice in resource-rich countries, such objective measures of assessment are not widely available in LMICs like Pakistan [74, 75], and national dysphagia assessment guidelines for SLTs are unavailable. Some Pakistani studies have recommended upper gastric intestinal endoscopy to assess dysphagia [74–77], however the diagnostic criteria are vague and inconsistent, with some adverse effects noted, and studies do not report much SLT input. Furthermore, accessibility to and affordability of the procedure in Pakistan limits its use in clinical practice. Considering these factors, and that services provided are community based, knowledge of observational signs and symptoms may provide guidance to SLTs in Pakistan for observational clinical exams of dysphagia. Awareness of rehabilitation in Pakistan is still in its infancy [78], and despite SLTs being part of rehabilitation teams, there remains a substantial lack of awareness surrounding the need for SLTs. At present, there are no known studies on management of dysphagia by SLTs in Pakistan, nor is information available regarding support groups and community referrals.

Thus, there is an urgent need to determine current practice within Pakistan, and subsequently determine feasible, accessible, and clinically efficient tools to appropriately assess dysphagia, informing suitable management approaches to improve patient outcomes [23, 25, 79]. This is particularly important since HICs’ approaches are limited in their applicability and generalisability to LMICs. Therefore, the current study aimed to:

- Explore the clinical practices of SLTs in Pakistan in the assessment, and management of dysphagia for both children and adults,
- Explore dysphagia signs and symptoms assessed by SLTs in Pakistan,
- Explore dysphagia service delivery and,
- Identify factors that both hinder and facilitate SLTs to assess and manage dysphagia in Pakistan.

## Method

### Survey Design and Development

A cross-sectional online survey with 45 items was designed to gather data on the clinical practices of SLTs working with children (0–18 years) and adults with dysphagia in Pakistan. Development and reporting were informed by published guidelines from the Checklist for Reporting Results of Internet E-Surveys (CHERRIES) [80] *(see Supplementary Material),* with no participant incentives offered*.* The questions were adapted for the Pakistani setting from a previous 47-item survey designed by Howells et al. [32] for Australian SLTs. Some original survey questions were removed as they were considered less relevant for this study’s aims (e.g., patient waitlists, prioritization, and discharge criteria) [32]. Given the exploratory nature of this research, additional questions were added to specifically investigate signs and symptoms observed by SLTs in Pakistan during CSEs, perceptions of own competence, and to incorporate dysphagia practices with children. The questions on signs and symptoms of dysphagia were developed using prior literature for both children and adults [45–51], and included items that had less evidence (e.g., nasal congestion/stuffy nose), since the research team judged that they may be in clinical usage despite this.

The initial draft was created by the research team who were all practicing SLTs. Two members of the team (NB, SM) had over 20 years’ clinical experience in working with either children or adults in HICs. The third member of the team (RA) had approximately 10 years’ experience working with both populations in Pakistan. The survey was created using Qualtrics (www.qualtrics.com/uk), a secure, anonymised online platform. The questionnaire was then piloted with a group of four SLTs; one had disseminated a similar survey in the UK previously, and three were practicing SLTs in Pakistan. Based on their feedback, only minor changes regarding rephrasing of a few questions were suggested. The complete survey can be found in the *Supplementary Material.* The survey’s final version comprised 45 questions, addressing the following areas:

1. Consent, eligibility to participate and demographics (12),
2. General clinical practice (3),
3. Dysphagia-specific practice for both adults (8) and children (8),
4. Collaboration and teamwork practices (2),
5. Patient/Caregiver education (2),
6. Outcome measurement (4),
7. SLTs self-education practices (2) and,
8. Service evaluation (4).

Response formats included binary and multiple choice, rating Likert scales (e.g., never, occasionally, often, always), and open-ended text boxes where closed responses were not appropriate or additional insight would be useful. The overall length of the survey was measured to make participation optimal, with display and skip logic functions utilized to decrease participant exposure to irrelevant items (e.g.: not asking questions about children if the therapist did not work with this population as part of their caseload). Carryover choices were also utilized to reduce repetition and increase personalisation of responses.

### Participants and Procedure

Participants were qualified SLTs working with IWD in various settings. Participants also included 3rd and 4th year student SLTs who were involved with IWD, since they are commonly engaged in supervised dysphagia practice, and considered part of the Pakistani SLT workforce.

Inclusion criteria for participants included.

(1) Practicing SLTs or Student SLTs in 3^rd^ or 4^th^ year of a bachelor’s/master’s programs working in Pakistan, (2) Engaged in dysphagia practice.

Exclusion criteria included.

(1) Student SLTs in 1^st^ or 2^nd^ year of a bachelor’s program in Pakistan, (2) SLTs not working in Pakistan, and (3) SLTs not engaged in dysphagia practice.

An open invitation was sent via the primary investigators professional networks to recruit participants from Pakistan through email, social media (e.g., Facebook) and text/WhatsApp messaging, which is a well-established, preferred, and common networking method used by SLTs in Pakistan. Snowball sampling was used, where recipients were requested to forward the survey link on to other SLTs in their own circles to help increase reach and participation. By targeting a variety of platforms, we aimed to capture participants working in public or private settings and representing a range of geographical locations. Thus, the response rate was not calculated due to the recruitment strategy utilized. The survey took approximately 15 min to complete and all participants who wished to participate clicked the survey link.

Ethical permission for this study was obtained through the School of Health Sciences, City University of London (Ref: ETH 1920–1369). The questionnaire was available for four weeks during July 2020 and redistributed half-way through the active period of the survey. All data stored were anonymised and no identifiers were collected, including IP addresses, to protect the identity of the participants.

### Data Analysis

The data were downloaded from Qualtrics into a Microsoft Office 365 © Excel spreadsheet. Participants who declined consent and did not proceed with the survey questions beyond demographics (i.e., Q. 12) were excluded. Descriptive analysis of the responses was performed through Statistical Package for Social Sciences version 27 and included frequencies, proportions, and graphs, to allow for visual inspection of the data. Open-text responses were analyzed by the first author using conventional content analysis described by Hsieh & Shannon [81] to identify codes and categories generated directly from the responses. The coded data were then checked independently by a second member of the research team (NB) to verify the analysis. The datasets generated during the current study are available from the corresponding author on reasonable request, except data which contains identifying information.

## Results

### Participants

In total, 245 participants accessed the survey, of which six declined to participate, 137 did not meet the eligibility criteria, and one participant completed demographics but did not proceed further. In the final analysis, 101 participants were included. Since all participants did not complete all questions, results are reported as percentages and number values representing those who attempted each question.

The demographic profiles included in the final analysis are summarized in Table 1. Overall, the participants were largely female (93.1%), and engaged in full-time work (61.4%). More than half (64.4%) had clinical experience of five years or less, and over three quarters (79.2%) were involved in dysphagia practice for five years or less.

**Table 1:** Participant demographics (n = 101)

Age Interval	n	%
20–30 years	74	73.3
31–40 years	13	12.9
41–50 years	12	11.9
51–60 years	2	2.0
> 61 years	0	0.0
Sex		
Male	7	6.9
Female	94	93.1
Other	0	0.0
Clinical Experience		
Student SLT	14	13.9
2 years or less	24	23.8
5 years or less	27	26.7
6–10 years	27	26.7
11–15 years	5	5.0
> 15 years	4	4.0
Dysphagia Experience		
2 years or less	54	53.5
5 years or less	26	25.7
6–10 years	17	16.8
11–15 years	4	4.0
> 15	0	0.0
Dysphagia Caseload %		
5% or less	43	42.6
6–10%	20	19.8
11–30%	13	12.9
31–50%	13	12.9
51–75%	10	9.9
> 75%	2	2.0
Region		
Urban	60	59.4
Rural	15	14.9
Both	26	25.7
Work Setting (multi-select item)		
Acute hospital setting	53	24.0
Inpatient rehabilitation setting	47	21.3
Outpatient setting or community setting	75	33.9
School (including Special Education centers/nurseries)	30	13.6
Non-government Organization	16	7.2
Predominant Setting (if more than one selected)		
Acute hospital setting	16	25.0
Inpatient rehabilitation setting	9	14.1
Outpatient setting or community setting	32	50.0
School (including Special Education centers/nurseries)	6	9.4
Non-government Organization	1	1.6
Working with Adults/Children		
Children	23	25.7
Adults	9	8.9
Both	66	65.3

### General Clinical Practice

Results showed that some participants only saw children (25.7%) or adults (8.9%), while the majority saw both (65.3%). Clients were often seen 2–3 times a week (52.5%) and were commonly referred by allied health workers (32.8%), doctors (34%) or self-referrals (30.5%).

### Dysphagia Assessment Procedures

Results showed that SLTs were working with a variety of pediatric and adult disorders; most commonly reported pediatric disorders were developmental (19.4%), cerebral palsy (19.4%), and congenital (15.1%). Commonly reported adult disorders were stroke (16.2%), acquired brain injury (15.0%), and Parkinson’s and other progressive diseases (13.4%).

Assessment procedures (see Table 2) also varied and SLTs most frequently relied on CSEs to assess dysphagia in both children (34.2%) and adults (30.8%). Other instrumental tools and methods used were also reported including, FEES, VFSS, cervical auscultation, cough reflex testing and oximetry. The results also revealed that participants with more general and dysphagia-specific experience reported more use of instrumental assessments, compared to participants with less experience.

**Table 2:** Dysphagia assessment practices in children and adults

Measures used to assess dysphagia (multi-select item)	Children (*n* = 73)		Adults (*n* = 56)	
	n	%	n	%
Fibreoptic endoscopic evaluation of swallowing	28	17.4	27	17.3
Videofluoroscopic swallowing study	24	14.9	28	17.9
Clinical feeding evaluation	55	34.2	48	30.8
Cervical auscultation	12	7.5	14	9.0
Pulse oximetry	7	4.3	5	3.2
Cough reflex testing	31	19.3	30	19.2
Other (e.g., ‘Four finger test’)	4	2.5	4	2.6

The dysphagia indicators SLTs measured most in children were choking (100.0%), gagging (100.0%), and coughing (95.8%), whereas stuffy nose (70.4%) and drop in oxygen saturation (*n* = 33; 60.0%) were measured least. In adults, participants measured coughing (*n* = 54; 98.2%), voice quality changes (*n* = 52; 98.1%) and food residue (*n* = 51; 98.1%) most, and hypoxia (*n* = 26; 60.5%) and fever (*n* = 23; 56.1%) were among the least measured.

## Current Dysphagia Management Practice in Children and Adults

Overall, most commonly used management practices were a combination of compensatory strategies and active rehabilitation for both children (76.7%) and adults (80.8%). SLTs reported that they modified food (64.5%) and fluid (75.3%) in children, and food (38.0%) and fluid (40.0%) in adults for at least half their caseloads. A majority of respondents (62.4%) reported a dysphagia caseload of under or around 10%. The authors examined the data and found no significant differences between these practitioners and those with higher dysphagia caseload in the signs and symptoms they were observing in clinical swallow examinations. No significant differences were found for practices of SLTs with more experience compared to SLTs with 2 years or less experience. However, a statistically significant association was found between increased years of dysphagia experience and greater perception of providing best practice (*p* = 0.009). Further, most participants reported using the Free Water Protocol [61] for selected clients only, both in children (68.5%) and adults (60.7%). Increased usage was reported by participants with dysphagia experience of 2 years or more. Almost all participants reported that they always or often use individual sessions as the preferred service format for both children (95.9%) and adults (92.9%).

### Psychosocial Outcomes

Regarding psychosocial support, 68% SLTs said they refer patients and caregivers to support groups, while 4% did not, and over a quarter (28%) said such groups were unavailable. Almost all participants stated they always or often (90.7%) involve caregivers in their sessions, whereas a few said they occasionally or never do (9.3%).

In addition, 73 participants responded to the open-ended question regarding tools used for psychological outcome measurement in IWD, including functional impact (82.2%) and quality of life (79.5%). Informal methods were most often utilized, such as interviews, patient- and family reports, self-developed checklists, and clinical observations. Participants also reported using the SWAL-QOL [82]. Wellbeing was also routinely measured by approximately half of the SLTs for clients (54.8%) and less for caregivers (39.7%).

### Factors Affecting Service Delivery

The open text responses described a range of facilitators that impact delivery of dysphagia services to children and/or adults. Participants reported that a supportive and multidisciplinary team, and access to current evidence and resources were important: *‘Neurorehab MDT with weekly meetings and discussions,’ ‘availability of resources and supportive team’* and *‘collaboration practices with other physicians such as nutritionists*.’

Other factors that facilitate service delivery included, motivated and supportive clients that made consistent progress: *‘progress and positive feedback from the clients.’* Participants highlighted further facilitators such as being self-motivated and current with the evidence base; and exposure to and experience with individuals with dysphagia: *‘passion to work,’ ‘staying up to date with latest research,’ ‘my own determination to work beyond lines and pays’* and *‘my vast exposure with dysphagia patients with many supervisors in different settings and continuing education.’* Participants were able to indicate a range of ways to keep updated with current dysphagia practices including, textbooks, research articles, online and in-person training courses and/or conferences, YouTube and social media (such as Instagram and Facebook): *‘the internet at my disposal to independently research the best management techniques for my client.’*

The responses gathered about barriers revealed a wider issue with awareness of and motivation to manage dysphagia (both from the perspectives of SLTs and family). For SLTs, there was a lack of evidence-based knowledge, and guidance from within the workplace: *‘Lack of exposure and guidance,’ ‘there aren’t many continuing professional development courses in Pakistan’* and *‘limited evidence-based practice.’* For the family, respondents reported a lack of knowledge which may lead to problems with consistency, compliance and not viewing dysphagia therapy as a priority: *‘lack of motivation and carrying over the activities at home.’* Other issues are related to reduced literacy and access to services due to the geographical location of patients. More broadly, limited knowledge and awareness of the SLT role by other professionals was reported, which may impact referrals and involvement of SLTs: *‘No referrals from doctors,’ ‘physicians lack of knowledge and limited confidence in SLT expertise,’* and *‘poor communication with nursing homes*.’ There was also a lack of resources including, patient handouts, evaluation methods and SLT time to conduct dysphagia assessments: *‘Shortage of assessment tools,’ ‘unavailability of resources in Urdu,’ ‘large caseload,’* and *‘allied health professionals work only in urban areas these facilities are not available in rural areas.*

## Discussion

This study aimed to elucidate and increase the understanding of SLTs’ dysphagia practices in Pakistan, with emphasis on assessment, management, and service implementation. Dysphagia practices have been examined by researchers in HICs [32, 83–85], and dysphagia has been clinically investigated in Pakistan [70–76]. To the best of the research team’s knowledge this is the first nationwide survey focusing on dysphagia care by SLTs in this geographic setting and provides an important first exploration into the area. Our results revealed variability in assessment and management processes, which is consistent with previous clinical practice survey findings, including a study from India, another LMIC [86].

Our findings suggest that SLTs perform multiple roles, including educational, advocacy, counseling, and treatment. While the sample size was lower than surveys from HIC [32, 83, 84], this was expected as SALT is less established in Pakistan, and awareness and prioritization of allied health professions is far less in comparison [78]. A survey assessing dysphagia practices in India [86] was similarly affected by the smaller number of SLTs in the country. Khan et al. [87] estimated a total of 250 qualified SLTs in Pakistan in 2015 which makes the participants in this study a significant proportion of the total practicing SLTs, increasing our confidence in the representativeness of the participants. The findings additionally support generalisability as the sample is representative of SLTs across Pakistan, with a range of clinical and dysphagia experiences, and a variety of settings and clinical populations. When considering experience levels, in some Australian studies approximately half of the SLTs had at least 5 years’ experience of dysphagia [83], while Howells et al. [32] reported a median of 9 years for their sample. Most participants in our sample had lower general and dysphagia-specific experience, which was also true for neighboring LMIC India [86]. This finding is not entirely unexpected as the profession is relatively new in Pakistan, similar to India, with only a few training programs, and a few rehabilitation teams that include SLT services [78, 86]. This further reinforces the notion that there is a pressing need for understanding and promoting SLT within Pakistan. For example, these results suggest that the majority of SLTs work in private facilities in urban settings, and a quarter work in both rural and urban areas. This indicates an insufficient coverage of country-wide services since approximately 63% of Pakistanis live in rural or remote areas [88].

Referral levels to SLTs were similar from self-referrals, and other professionals, in contrast to evidence from other countries [32, 83]. Self-referral is not uncommon in Pakistan due to privatized healthcare, but referral rate might vary depending on the context the SLT works in, such as rural or urban, private, or public, and hospital or community. Dysphagia contributed to a small percentage of the total caseload for nearly half of Pakistani SLTs, consistent with SLTs in India [86], whereas dysphagia constituted more than 50% of the caseload for almost half the respondents in a Canadian survey [83]. Such a finding is concerning given the prevalence of dysphagia and its consequences in a LMIC like Pakistan. Most SLTs in this survey also worked with both children and adults, which may present a challenge to stay current with practice guidelines for caseloads that are likely to be heterogenous in age and diagnosis.

Regarding assessment procedures, most SLTs relied on CSEs for dysphagia, congruent with earlier HIC and LMIC surveys [32, 83, 84, 86]. CSE usage is likely to be higher because those who selected ‘other’ also listed informal clinical exams such as ‘bedside swallow evaluation’ and ‘informal assessment’. While it is encouraging to see that instrumental methods are used by approximately 15% of SLTs, their feasibility in this LMIC is questionable due to reduced affordability and accessibility [74]. Service limitations described by participants included limited availability of instrumental evaluation and trained staff, as well as reduced awareness of dysphagia in patients and physicians, all of which threaten the applicability of these tools to resource diverse settings within Pakistan.

Variability was seen in symptom identification which is concordant with past literature and was to be expected [89]. This can be attributed to dysphagia severity and SLT education/training level. Variations in clinical practice were further underlined by reduced measurement of wellbeing outcomes, which may be related to lack of time as a result of busy caseloads, as well as reduced recognition, training, and skill level. Earlier physician-centered studies in Pakistan have used instrumental evaluation to examine dysphagia symptoms but without collaborating with SLTs [74–77]. The current study’s participants described a lack of collaboration between SLTs and physicians, matching the previous research. Findings herein may encourage collaborative care between SLTs and physicians to provide multidisciplinary diagnostic services to IWD, thereby improving patient outcomes.

Interestingly, a high number of participants reported using the citric acid cough reflex testing assessment, despite it being unavailable in Pakistan. The question may have been misunderstood as assessing coughing ability alone (e.g.: asking patient to cough). The Free Water Protocol [61] was reported as a management strategy for specific clients by many participants. Although there is insufficient high-quality evidence to support that it does not cause respiratory complications, the use of this protocol for strictly selected adult clients may be useful, and enhance patients' perceptions of quality of life [62]. This level of use is comparable with Howells et al.’s [32] survey, and may highlight how the protocol is used in both HIC and LMIC settings. However, over three quarters of participants reported using this protocol in children despite a lack of evidence. This implies an urgency to explore clinical knowledge and practice within Pakistan further and supports the establishment of clinical guidelines.

Treatment for dysphagia included preference for individual sessions, and prescription of compensatory techniques alongside active treatment, irrespective of age. Over half the participants saw the same patients weekly or more often. While regular individual sessions may be considered intensive and useful, this model may deprive patients of the benefits of group therapy. Additionally, consistency modification was utilized more often in children than in adults, however the efficacy and long-term effects of this approach remain unclear for both children and adults [52, 55–60, 84]. Furthermore, psychosocial groups may improve wellbeing outcomes of IWD and although most SLTs encouraged support groups, not many psychosocial support systems are available in Pakistan, and would need to be explored in the future.

Another aim of this study was to explore factors that influence service delivery. SLTs described reduced awareness among physicians and families, limited resources, and training opportunities, all of which impact quality of care. Barriers were frequently service- or client-related, e.g., *‘limited resources*,’ whereas facilitators were more participant-related, e.g., *‘my motivation.’* This judgment of value may be attributable to the importance SLTs attach to their own profession, or participants’ self-reliance to enhance service delivery due to inadequate wider support and recognition.

Further, clinical implications of these findings include, but are not limited to, further development and support of the Pakistani SLT workforce, e.g., increase awareness of evidence-based assessment and management options being utilized across the country (such as citric acid cough reflex testing and free water protocol), recognition of psychosocial needs and measurement of such outcomes. In addition, support to encourage collaborative and trans-disciplinary care, e.g., campaigns to increase physician awareness of SLT practices, activities to address barriers to service and providing support, and systemic changes to enhance clinical standards for dysphagia care. Early identification of dysphagia symptoms for patients in Pakistan will lead to prompt and effective early intervention, which can mitigate negative long-term outcomes.

### Limitations

This study’s results must be interpreted in the LMIC context and context of methodological limitations. The recruitment method was restricted to the primary investigator’s professional network and of those contacted. There is a possibility of selection bias, omission of those in remote regions with limited internet connectivity, and inclusion bias based on clinical interest. Hence, the applicability of the findings may be restricted to those in better-resourced contexts. Despite this, many participants from rural areas did participate including those who had spent limited clinical time on dysphagia. Additionally, the survey was conducted in English. Over half of those that provided consent did not select both options to proceed past the eligibility criteria in question 2. This may have meant that they were ineligible to participate, or potentially indicates an issue with survey usability or readability, and/or individual participant proficiency levels. This may decrease the validity of our findings. However, Pakistan’s official language is English, all known SLT programs are taught in English, and this issue was not noted during the pilot phase with Pakistani SLTs. The survey was only accessible for one month which may also have reduced the response rate. Self-reported surveys may lead to recall and self-report bias. Under- or over-estimation of caseload proportions or own skill level by participants is also possible since these are self-calculations and not confirmed values. Also, these are service provider perspectives, and a bias is observed against patients and caregivers, thus subsequent research with multiple sources of data can help distinguish between actual and perceived barriers. Data regarding socio-geographic factors collected in international surveys, e.g., city of practice, were not collected in this study. This information could have provided further insight into the spread of SLT services.

Moreover, the questionnaire itself may have potential limitations since it was adapted from an Australian survey by Howells et al., [32]. Pakistan’s LMIC setting differs from the Australian HIC context and modifications made for this research will have affected validity. On balance, some question items did not have sufficient research evidence-base to include them, for example free water protocol use with children. Some open-ended responses were short and could have caused interpretation bias, e.g., the answer ‘consistency’ was ambiguous and could have been interpreted as consistency of treatment, food/fluid, home-practice, or regularity of sessions. However, as mentioned, ambiguities were not detected during the pilot. Finally, findings may not generalize to other countries with different healthcare structures but are likely to be relevant to other LMICs and settings with privatized healthcare systems.

Despite the limitations, this research was valuable in providing novel data on an under-studied workforce and disorder population in Pakistan, and providing useful information with many strong positives, e.g., representative group of SLTs surveyed. However, it also raises questions that need to be addressed, e.g., why some participants have reported use of assessments they are unlikely to have access to (citric acid cough reflex testing). Although there were methodological concerns, over 100 people participated in this survey making it the largest and only SLT survey at present in Pakistan, increasing our confidence in the applicability of the findings.

### Future Directions

This research enables recommendations for future investigations as much remains unexplored. Evidently, further focus must be placed on physician awareness, perspectives of service users, inclusion of caregivers in the narrative, and provision of psychosocial support for IWD. There is currently no robust population‐specific evidence on dysphagia, or its management in Pakistan. Longitudinal studies should be carried out to track changes in swallowing functions in varying disorder populations in the country. Collaborations with physicians for research will provide novel data, and reasons for under-diagnosis and under-referral may become clearer. Additionally, collaborating with mental health professionals and providing them with specialized training on dysphagia and its effects may address the need for psychosocial support and support groups. This project aimed to understand current clinical practices of SLTs, and it was successful in doing so, but until these are fully understood from all perspectives, service provision will be less than optimal. Lastly, insight gathered through this research may effectuate the development of clinical guidelines for SLTs and encourage collaborative care to maximize treatment efficacy and benefits for patients.

## Conclusion

This exploratory research provides a preliminary picture of dysphagia care by SLTs in Pakistan, working with heterogenous populations, settings, and resources. It starts to address a significant gap in research in LMICs, an under-studied area, and highlights an urgent need for increased awareness and support of dysphagia services in Pakistan. Findings suggest that a small proportion of SLTs are managing dysphagia, often alongside other clinical practice areas. Informal clinical exams are largely used for assessment, and the emerging use of instrumental techniques is promising. Barriers and facilitators to service delivery described provide a framework for improvement. Further provision of services and support is of heightened importance to decrease consequences and improve patient outcomes, particularly in LMICs. Information from this research has established the groundwork to improve knowledge of SLTs and other health professionals engaged in dysphagia practice, thereby establishing efficient care pathways in Pakistan, and improving overall quality of services.

### Supplementary Information

Below is the link to the electronic supplementary material.

Supplementary file1 (PDF 83 KB)

Supplementary file2 (PDF 210 KB)
