# Supplementary material for: Understanding Dysphagia Care in Pakistan: A Survey of Current Speech Language Therapy Practice
Source: Dysphagia. 2023 Nov 25;39(3):484–94. doi: 10.1007/s00455-023-10633-7 (PMC11127846; doi:10.1007/s00455-023-10633-7)
Supplement: Supplementary file 1 — Supplementary file1 (PDF 83 KB) [file 455_2023_10633_MOESM1_ESM.pdf]

Supplementary Material 1

Checklist for Reporting Results of Internet E-Surveys (CHERRIES) (Eysenbach, 2004)

**Item category**	**Checklist item**	**Y/N**	**Comments**
Design	Describe survey design	Y	The survey was mixed-method design with MCQ/BINARY/Likert scales and open-text boxes. Target population is described in method section. Snowball sampling was used where Speech language therapists (SLTs) were asked to forward the survey link to colleagues and other SLTs.
Institutional Review Board (IRB) approval and informed consent process	IRB approval	Y	Ethics approval was acquired through School of Health Sciences, City University of London - (REC reference number: ETH1920-1369)
	Informed consent	Y	Participant information sheet was embedded into the survey form. Consent was provided explicitly by ticking the option 'I wish to participate', and by virtue of completing the survey.
	Data protection	Y	Only the research team had access to password-protected data on Qualtrics. No personal information was collected or stored.
Development and pre-testing	Development and testing	Y	As described in method section.
Recruitment process and description of the sample having access to the questionnaire	Open survey vs closed survey	Y	Open survey
	Contact mode	Y	Contact with potential participants was made through electronic and social media (e.g., mailing lists and Facebook) of primary investigator, and text-messaging applications (e.g. WhatsApp) as described in method section.
	Advertising the survey	Y	
Survey administration	Web/E-mail	Y	Web
	Context	Y	As described in the method section.
	Mandatory/voluntary	Y	Voluntary
	Incentives	Y	No financial incentives offered. One-page summary of results was offered if email addresses were given.
	Time/Date	Y	28-06-2020 to 28-07-2020
	Randomisation of items or questionnaires	N	
	Number of items	Y	45, including consent question, eligibility criteria and email address items.
	Number of screens (pages)	Y	11 pages (including participant information and eligibility criteria pages)
	Completeness check	Y	Forced-response feature chosen on Qualtrics
	Review step	Y	Respondents were able to review and change their answers.
Response rate	Unique site visitor	N	Collection of IP addresses and cookies were disabled to protect anonymity of respondents.
	View rate (Ratio of unique survey visitors/ unique site visitors)	N	
	Participation rate (Ratio of unique visitors who agreed to participate/	N	

	unique first survey page visitors)		
	Completion rate (Ratio of users who finished the survey/users who agreed to participate)	Y	65/101 x 100 = 64.4%
Preventing multiple entries from the same individuals	Cookies used	N	Cookies were not used to assign unique identifiers to users in light that some participants may drop out and want to start a survey again.
	IP check	N	IP addresses were not collected to protect the identity of respondents.
	Log file analysis	N	
	Registration	N	Open survey was used
Analysis	Handling of incomplete questionnaires	Y	Responses terminated beyond demographic section were included in final analysis with completed surveys. Only responses that terminated before completing demographic section were excluded.
	Questionnaires submitted with an atypical timestamp	N	
	Statistical correction	N	None since a representative sample was collected
