# Supplementary material for: Understanding Dysphagia Care in Pakistan: A Survey of Current Speech Language Therapy Practice
Source: Dysphagia. 2023 Nov 25;39(3):484–94. doi: 10.1007/s00455-023-10633-7 (PMC11127846; doi:10.1007/s00455-023-10633-7)
Supplement: Supplementary file 2 — Supplementary file2 (PDF 210 KB) [file 455_2023_10633_MOESM2_ESM.pdf]

CITY
UNIVERSITY OF LONDON
EST 1894

Supplementary Material 2

Understanding Dysphagia Care in Pakistan: A Survey of Current Speech Language Therapy Practice

S/no	Question	Response
**Section 1: Participant Information and Demographics**		
1.	Participant Information Statement	I wish to participate<br>I do NOT wish to participate [skip to end of survey if selected]
2.	I am a qualified or student speech and language therapist who:	(a) has worked with at least one client in the last year with Dysphagia; and<br>(b) has worked in an acute, inpatient rehabilitation, outpatient hospital setting, or community/private setting.<br>If yes to all two points, please click here to continue.<br>If no, please click here. [skip to end of survey if selected]
3.	My age is:	20-30 years<br>31-40 years<br>41-50 years<br>51-60 years<br>61+ years
4.	I identify as	Male<br>Female<br>Other
5.	I have been working as a SLT/involved in clinics for	Student clinician<br>Less than 2 years<br>2-5 years<br>6-10 years<br>11-15 years<br>More than 15 years
6.	My experience working with Dysphagia is	Less than 2 years<br>2-5 years<br>6-10 years<br>11-15 years<br>More than 15 years
7.	In my current role/most recent previous role in which I worked with people with Dysphagia, the approximate percentage of my caseload that includes patients with Dysphagia is:	5% or less<br>6-10%<br>11-30%<br>31-50%<br>51-75%<br>More than 75%
8.	I am currently working	Full time<br>Part time<br>Student
9.	The region I currently work in is	Urban<br>Rural<br>Both
10.	I work in (select **all** that apply):	Private healthcare sector<br>Public healthcare sector<br>NGO
11.	I work in (select **all** that apply)	Acute hospital setting<br>Inpatient rehabilitation setting<br>Outpatient setting or community setting (e.g. private clinics)<br>School (including Special Education centres/nurseries)

		NGO
12.	In the previous question, you have indicated that you work in multiple settings. Please select the setting that you predominantly work in	Acute hospital setting<br>Inpatient rehabilitation setting<br>Outpatient setting or community setting<br>School (including Special Education centres/nurseries)<br>NGO
**Section 2: General Clinical Practice**		
13.	I work with (select **all** that apply)	Children<br>Adults<br>Both
14.	Please indicate who may refer a client to your service: (select **all** that apply)	Client initiated (self-referral)<br>Medical doctors<br>Allied health professionals (including other SLTs)<br>Other _______
15.	In general, I see patients	Daily<br>Once a week<br>2-3 times per week<br>Fortnightly (once in 2 weeks)<br>Monthly<br>Other: _____
**Section 3a: Dysphagia Specific Practice (Children)**<br>[Display this section if 'children' or 'both' is selected in Q 13]		
16.	Of the children I see with dysphagia, the client groups are (select **all** that apply):	Developmental conditions (e.g. intellectual disability. Down and other syndromes)<br>Congenital conditions (e.g. cleft lip/palate, craniofacial conditions)<br>Cerebral Palsy<br>Complex medical and medically fragile conditions (including prematurity)<br>Autism and Avoidant/Restrictive Food Intake Disorder (ARFID)<br>Unknown aetiology<br>Other neurological (e.g. Spinal muscular atrophy, childhood stroke)<br>Other: _______
17.	When managing clients with dysphagia, which of the following service models do you adopt in your practice? (Please choose the appropriate response for each item)	(see table below)

	Never	Occasionally	Often	Always
Individual treatment sessions only				
Group & individual treatment sessions				
Group sessions only				

18.	What assessments or measures do you use to assess dysphagia in patients?	Fibre-endoscopic Evaluation of Swallowing FEES<br>Video Fluoroscopic Swallow Study/ Modified Barium (VFSS/MBS)<br>Clinical Swallow Exam<br>Cervical Auscultation<br>Pulse Oximetry<br>Cough Reflex Testing<br>Other: _____
19.	What signs and symptoms do you typically look for in children?	(see table below)

Sign/Symptom<br>(Weir et al., 2009, DeMatteo et al., 2005, Arvedson 2006,, Lefton-Greif and McGrath-Morrow, 2007)	Yes	No
Multiple swallows		
Vomiting		

<table>
<tr>
<td></td>
<td></td>
<td>
<table>
<tr><td>Cough during feeding</td><td></td><td></td></tr>
<tr><td>Wet or gurgled breathing</td><td></td><td></td></tr>
<tr><td>Wet voice</td><td></td><td></td></tr>
<tr><td>Wheezing or rattling</td><td></td><td></td></tr>
<tr><td>Stridor</td><td></td><td></td></tr>
<tr><td>Stuffy nose</td><td></td><td></td></tr>
<tr><td>Eye tearing</td><td></td><td></td></tr>
<tr><td>Clearing of throat</td><td></td><td></td></tr>
<tr><td>Choking</td><td></td><td></td></tr>
<tr><td>Gagging</td><td></td><td></td></tr>
<tr><td>Difficulty in breathing while feeding</td><td></td><td></td></tr>
<tr><td>Temperature spikes within 24 hours of feeding;</td><td></td><td></td></tr>
<tr><td>Drop in oxygen saturation</td><td></td><td></td></tr>
<tr><td>Apnoea during feeding (halt in breathing or facial colour change/circumoral)</td><td></td><td></td></tr>
</table>
Other: ____
</td>
</tr>
<tr>
<td>20.</td>
<td>Which management approach do you predominantly use? (Please choose only one of the following):</td>
<td>Compensatory management only (e.g., postural, fluid/food modifications) with no use of active rehabilitation

Combination of compensatory and some rehabilitation / safe swallow techniques (e.g., mildly thick fluids with a supraglottic swallow technique)

Combination of compensatory and regular active rehabilitation (e.g., postural / dietary modifications with additional active rehab program of exercises and manoeuvres)</td>
</tr>
<tr>
<td>21.</td>
<td>How often would you recommend a Free Water Protocol for your clients with dysphagia? (Please choose only one of the following):

Preamble: The Frazier Free Water Protocol (Panther, 2005) detects patients who are at risk of aspiration on thin fluids and have been recommended to safely consume only thickened liquids or nothing by mouth (NPO). This is in addition to any fluid modification recommendations during meals. The developers of this protocol hypothesised that when water is aspirated, the chance of it causing pneumonia is reduced but other risk factors of aspiration pneumonia need to be managed.</td>
<td>Never
For selected clients only
Often
Always</td>
</tr>
</table>

<table>
<tr><td>22.</td><td>What approximate proportion of your clients with dysphagia require modifications of <u>FOOD</u> consistencies?</td><td>Less than 5%
Between 5-10%
Between 11-25%
Between 26-50%
Between 51-75%
More than 75%</td></tr>
<tr><td>23.</td><td>What approximate proportion of your clients with dysphagia require modification of <u>FLUID</u> viscosity?</td><td>Less than 5%
Between 5-10%
Between 11-25%
Between 26-50%
Between 51-75%
More than 75%</td></tr>
<tr><td colspan="3">Section 3b. Dysphagia Specific Practice (Adults)
[Display this section if ‘adult’ or ‘both’ is selected in Q 13]</td></tr>
<tr><td>24.</td><td>Of the adult clients I see with dysphagia, the client groups are (select all that apply):</td><td>Stroke
Acquired Brain Injury
Parkinson’s Disease or other progressive neurological conditions (e.g. motor neuron disease)
Dementia
Cancer
Psychogenic conditions
Developmental/congenital condition (e.g.: intellectual/learning disability, cerebral palsy, muscular dystrophy, etc.)
General aging
Unknown aetiology
Other _______</td></tr>
<tr><td>25.</td><td>When managing clients with dysphagia, which of the following service models do you adopt in your practice? (Please choose the appropriate response for each item)</td><td><table>
<tr><th></th><th>Never</th><th>Occasionally</th><th>Often</th><th>Always</th></tr>
<tr><td>Individual treatment sessions only</td><td></td><td></td><td></td><td></td></tr>
<tr><td>Group & individual treatment sessions</td><td></td><td></td><td></td><td></td></tr>
<tr><td>Group sessions only</td><td></td><td></td><td></td><td></td></tr>
</table></td></tr>
<tr><td>26.</td><td>What assessments or measures do you use to assess dysphagia in patients?</td><td>Fibre-endoscopic Evaluation of Swallowing FEES
Video Fluoroscopic Swallow Study/ Modified Barium (VFSS/MBS)
Clinical Swallow Exam
Cervical Auscultation
Pulse Oximetry
Cough Reflex Testing
Other:</td></tr>
<tr><td>27.</td><td>What signs and symptoms do you typically look for in adults?</td><td><table>
<tr><th>Sign/Symptom
(Forster et al., 2011, Sue Eisenstadt, 2010)</th><th>Yes</th><th>No</th></tr>
<tr><td>Dyspnoea (difficulty in breathing)</td><td></td><td></td></tr>
<tr><td>Fever</td><td></td><td></td></tr>
<tr><td>Tachypnoea (rapid breathing)</td><td></td><td></td></tr>
<tr><td>Delirium</td><td></td><td></td></tr>
<tr><td>Confusion</td><td></td><td></td></tr>
<tr><td>Pleuritic chest pain</td><td></td><td></td></tr>
<tr><td>Wheezing</td><td></td><td></td></tr>
</table></td></tr>
</table>

28.		Hypoxia (drop in oxygen saturation)<br>Cough<br>Weight loss<br>Sputum<br>Rales (rattling sound while breathing)<br>Change in functional status<br>Prolonged chewing and prolonged mealtimes<br>Food remnants in the oral cavity<br>changes in vocal quality (e.g. wet voice)<br>Drooling during meals<br>Nasal regurgitation<br>Sensation of blockage during swallowing<br>Other:
28.	Which management approach do you predominantly use? (Please choose **only one** of the following):	Compensatory management only (e.g., postural, fluid/food modifications) with no use of active rehabilitation<br><br>Combination of compensatory and some rehabilitation / safe swallow techniques (e.g., mildly thick fluids with a supraglottic swallow technique)<br><br>Combination of compensatory and regular active rehabilitation (e.g., postural / dietary modifications with additional active rehab program of exercises and manoeuvres)
29.	How often would you recommend a Free Water Protocol for your clients with dysphagia? (Please choose **only one** of the following):<br><br>Preamble: The Frazier Free Water Protocol (Panther, 2005) detects patients who are at risk of aspiration on thin fluids and have been recommended to safely consume only thickened liquids or nothing by mouth (NPO). This is in addition to any fluid modification recommendations during meals.<br>The developers of this protocol hypothesised that when water is aspirated, the chance of it causing pneumonia is reduced but other risk factors of aspiration pneumonia need to be managed.	Never<br>For selected clients only<br>Often<br>Always
30.	What approximate proportion of your clients with dysphagia require modifications of <u>FOOD</u> consistencies?	Less than 5%<br>Between 5-10%<br>Between 11-25%<br>Between 26-50%<br>Between 51-75%<br>More than 75%
31.	What approximate proportion of your clients with dysphagia require modification of <u>FLUID</u> viscosity?	Less than 5%<br>Between 5-10%<br>Between 11-25%<br>Between 26-50%<br>Between 51-75%<br>More than 75%

**Section 4. Collaboration Practice**		
32.	Do you encourage your clients with dysphagia and their families to access support groups or social groups?	Yes<br>No<br>Unavailable
33.	How much do you involve caregiver/s in your dysphagia sessions?	Never<br>Occasionally<br>Often<br>Always
**Section 5: Patient/Caregiver Education**		
34.	What aids do you use to educate clients and caregivers?	(see table below)
35.	What resources do you provide your clients (e.g.: handouts, cookbooks, etc.)	Open ended
**Section 6: Outcome Measurement**		
36.	Do you routinely collect <u>functional impact</u> measures from clients/caregivers with dysphagia? (such as fatigue, participation, independence etc.)	Yes<br>No
37.	Do you routinely collect <u>quality of life (QOL)</u> measures from clients/caregivers with dysphagia?	Yes<br>No
38.	Please tell us which tool/s or process you use	Open ended
39.	Do you monitor for signs of anxiety/ depression in clients with dysphagia and their caregivers?	(see table below)
**Section 7: Speech & Language Therapist Education Practices (self)**		
40.	What resources do you rely on to stay up to date with current practices in dysphagia management?	Open ended
41.	I would say my management of Dysphagia is consistent with best practice	Strongly agree<br>Agree<br>Neutral<br>Disagree<br>Strongly disagree
**Section 8: Service Evaluation**		
42.	The things that make it **difficult** for me to provide the best service to my Dysphagia patients are:	Open ended
43.	The things that **enable** me to provide the best service to my Dysphagia patients are:	Open ended

Question 34:

	Never	Occasionally	Often	Always
Visual information (e.g., diagrams, videos)				
Verbal information				
Written information				
Demonstration (e.g., of how to thicken fluids)				

Question 39:

	Clients	Caregivers
Yes, routinely		
Only if indicated		
No		

44.	Please leave any other comments that you may have about Dysphagia practices in Pakistan:	Open ended
45.	If you wish to receive a one-page summary of the results of this research, please provide your email address. Email addresses will not be stored or linked to your results to maintain confidentiality.	

Thank you for taking the time to respond.
